# Decreased Gray Matter Volume of Right Inferior Parietal Lobule Is Associated With Severity of Mental Disorientation in Patients With Mild Cognitive Impairment

**DOI:** 10.3389/fneur.2018.01086

**Published:** 2018-12-14

**Authors:** Ayame Oishi, Takao Yamasaki, Ayako Tsuru, Motozumi Minohara, Shozo Tobimatsu

**Affiliations:** ^1^Department of Neurology, Minkodo Minohara Hospital, Fukuoka, Japan; ^2^Department of Clinical Neurophysiology, Neurological Institute, Graduate School of Medical Sciences, Kyushu University, Fukuoka, Japan

**Keywords:** mild cognitive impairment, mental disorientation, Neurobehavioral Cognitive Status Examination (COGNISTAT), Mini-Mental State Examination, inferior parietal lobule, voxel-based morphometry, magnetic resonance imaging

## Abstract

**Background:** Mental disorientation in time, space, and with respect to people is common in patients with Alzheimer's disease (AD) and mild cognitive impairment (MCI). Recently, a high-resolution functional MRI (fMRI) study revealed that the inferior parietal lobule (IPL) and precuneus are important regions related to mental orientation in healthy individuals. We hypothesized that the IPL and/or precuneus are crucial regions for mental disorientation in patients with amnestic MCI (aMCI). Therefore, our aim was to assess our hypothesis in these patients using voxel-based morphometry (VBM).

**Methods:** Fifteen patients with aMCI participated. The Neurobehavioral Cognitive Status Examination (COGNISTAT) as well as the Mini-Mental State Examination (MMSE) were used to evaluate mental disorientation. Subsequently, we used VBM analysis to identify brain regions that exhibited gray matter (GM) volume loss associated with mental disorientation. Based on our hypothesis, four brain regions (bilateral IPLs and precuneus) were selected as regions of interest (ROIs).

**Results:** We found a significant decreased GM volume in the right IPL, which was correlated with lower orientation scores on the COGNISTAT. In contrast, GM volume in other ROIs did not show a significant positive correlation with mental disorientation. Regarding the MMSE, no significant reduction in GM associated with decline in orientation were observed in any ROI.

**Conclusion:** We found the significant relationship between low GM volume in the right IPL and severity of mental disorientation. Therefore, the right IPL is responsible for mental disorientation in aMCI.

## Introduction

Alzheimer's disease (AD) is a progressive neurodegener-ative disorder and the most common form of dementia in older people. It is characterized by numerous cognitive deficits including memory disturbance, disorientation, and visuospatial deficits (1, 2). In contrast, mild cognitive impairment (MCI) is an intermediate state between normal aging and dementia (3) and is classified into two subtypes: amnestic and non-amnestic MCI. Amnestic MCI (aMCI) is widely viewed as a preclinical stage of AD (4).

Orientation in time, space, and to people is fundamental for one's own behavior. Recently, a high-resolution functional magnetic resonance imaging (fMRI) study has revealed that common cortical activity related to orientation for time, space and people is mainly localized in the IPL and precuneus in healthy people (5). AD is a pathology known to preferentially involve temporo-parietal association areas, and this is true even in MCI (6). Nevertheless, which brain regions are associated with disorientation is still unclear in aMCI, even though disorientation is a major symptom.

Mini-Mental State Examination (MMSE) is the most commonly used tool for assessing cognitive functions including orientation to time (5 points) and space (5 points) for clinical and research purposes. However, it does not assess, orientation to people. Recently, the Neurobehavioral Cognitive Status Examination (COGNISTAT) has been introduced as a way to evaluate cognitive functions. COGNISTAT is a short cognitive battery that contains subtests for orientation to people (2 points), time (6 points), and space (4 points). Furthermore, AD patients exhibited significantly lower scores on many subtests of COGNISTAT compared with healthy older individuals (7). The total number of impaired scores on COGNISTAT is also useful for discriminating AD from non-AD dementia (7). Thus, COGNISTAT is likely a better tool than MMSE for studying the core brain regions associated with overall mental disorientation (including time, space, and people) in aMCI and AD.

Voxel-based morphometry (VBM) is a brain imaging method that can measure gray matter (GM) volume (GMV). The results can be used to assess the relationship between GMV and scores on neuropsychological tests related to various neurodegenerative disorders (8, 9). Therefore, VBM can reveal which brain regions are related to disorientation in aMCI and AD. The aim of this study was to use VBM to identify the brain regions associated with overall mental disorientation in aMCI using neuropsychological tests (COGNISTAT and MMSE). Based on the recent fMRI finding showing the important role of the IPL and precuneus on orientation in healthy humans (5), we tested the hypothesis that the IPL and/or precuneus are brain regions crucial for overall mental disorientation in aMCI.

## Materials and Methods

We analyzed data from the orientation subtests of COGNISTAST and MMSE (Japanese versions) and from high-resolution three-dimensional (3D) T1-weighted MRI images obtained from 15 aMCI patients who visited the Memory Clinic (outpatient dementia service) at the Minohara Hospital. The protocol for the present study was approved by the internal ethics review boards of Minohara Hospital.

### Patients

We retrospectively reviewed the medical records of 15 aMCI patients (9 females; age: 63–84 [mean: 75.3 ± 6.8] years; education: 9–16 [mean: 12.7 ± 2.0] years) from their first visit until September 2017. At the first visit, all patients underwent clinical neurological examinations by an experienced neurologist (T.Y.). Neuropsychological assessments were also performed by a clinical psychologist (A.T.), including the administration of COGNISTAT and MMSE, the delayed recall of logical memory on the Wechsler Memory Scale-Revised (delayed LM WMS-R), the Clinical Dementia Rating (CDR) scale, and the Geriatric Depression Scale (GDS). Furthermore, all patients completed electroencephalography, MRI, single photon emission computed tomography (SPECT), and standard laboratory tests. Inclusion criteria for aMCI followed the criteria of the Japanese Alzheimer's Disease Neuroimaging Initiative (10).

### MRI Acquisition and Analysis

T1-weighted 3D sagittal images were acquired using a 1.5-Tesla MRI scanner (MRT200PP3, Toshiba Medical Systems Corporation, Japan). The acquisition parameters were as follows; repetition time = 13.5 ms; echo time = 5.5 ms; flip angle = 20°; field of view = 220 mm; acquisition matrix = 256 × 256, and slice thickness = 1.5 mm.

T1 images were processed using the VBM in SPM12 (Functional Imaging Laboratory, University College London, UK) running on MATLAB R2015b (The Mathworks, Inc., USA). In the segmentation, registration, normalization and modulation process, we used default settings for all parameters but “Preserve” was changed from “Preserve Concentrations” to “Preserve Amount” for converting to volume. The images were segmented according to tissue type into GM, white matter (WM), and cerebrospinal fluid images (CSF), and non-tissue types (bones, soft tissue, and air). Then, the Diffeomorphic Anatomical Registration using Exponentiated Lie Algebra (DARTEL) tool box in SPM12 for registration, normalization, and modulation. DARTEL templates were created from all the present data (11). The registered images were transformed to Montreal Neurological Institute (MNI) space. Finally, the normalized and modulated images were smoothed with 8-mm full width half maximum Gaussian kernel and analyzed with SPM12 software. The volumes of the different tissue classes and the total intracranial volume (TIV, sum of GM, WM, and CSF) were calculated using a MATLAB script (http://www0.cs.ucl.ac.uk/staff/g.ridgway/vbm/get_totals.m).

Statistical analysis of GMV data was performed using SPM12. To test our hypothesis that the IPL and/or precuneus are important regions for disorientation in aMCI, four brain regions (bilateral IPLs and precuneus) were selected as regions of interest (ROIs). These ROIs were based on the anatomically defined ROIs in the WFU PickAtlas toolbox (12). For each ROI, small volume correction was performed (voxel-level FWE corrected, *p* < 0.05) with TIV as a covariate. Furthermore, for the peak MNI coordinate within each ROI, Pearson's correlation coefficient was calculated to evaluate the relationship between the GMV resulting from SPM12 analysis to extract raw voxel values within each ROI (13) and orientation scores on COGNISTAT and MMSE. We also adjusted the *p*-values to determine the effects of age, sex, and length of education for COGNISTAT and MMSE analyses, and additionally total MMSE score for COGNISTAT analysis. The difference of GMV loss associated with orientation scores between converters and non-converters were compared by the two-sample *t*-test. Further, a *post-hoc* statistical power analysis was conducted using G^*^power 3.1.9.2 [power (1-β)> 0.8 was generally acceptable] (14).

## Results

### Progression From MCI to AD

During the follow-up period (mean 19 ± 17 months), 10 of 15 patients who were diagnosed with aMCI converted to probable AD on the basis of medical records, neurological evaluation, or interviews.

### Neuropsychological Tests

The mean score on the orientation subtest of COGNISTAT was 10.6 ± 1.5 (full = 12, higher scores indicate better orientation). For the MMSE, the mean scores of total and orientation subtest were 25.8 ± 2.3 (full = 30) and 8.9 ± 1.1 (full = 10), respectively. The mean score on the Delayed LM WMS-R was 2.8 ± 2.3 (full = 25), and that for the GDS was 4.4 ± 3.1 (normal < 6). The mean CDR score was 0.5 (range = 0–3).

### VBM Analysis

**Figure 1:**
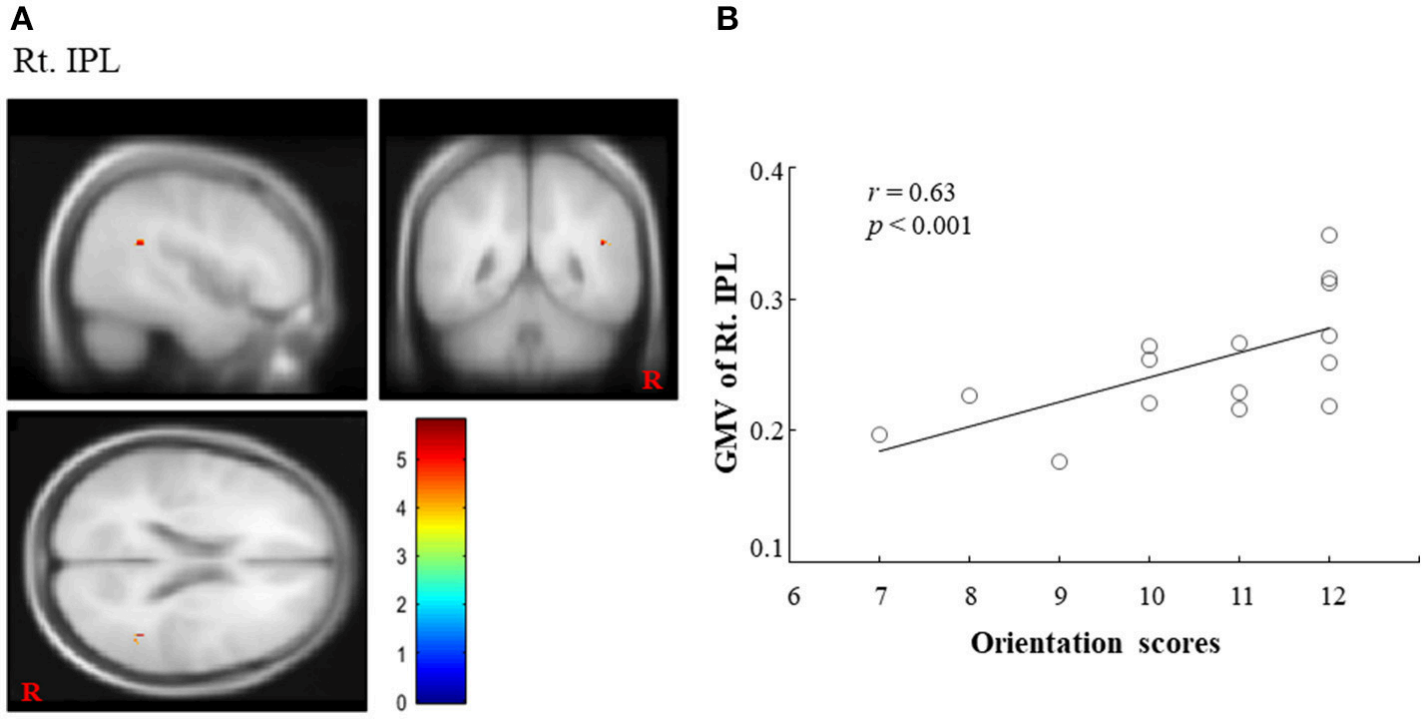
Importance of right IPL on COGNISTAT scores. **(A)** A regional cluster is shown in the right IPL (voxel-level FWE corrected, *p* < 0.05). **(B)** We found a significant positive correlation between the GMV in the right IPL and overall orientation scores on the COGNISTAT. IPL, inferior parietal lobule; GMV, gray matter volume; COGNISTAT, Neurobehavioral Cognitive Status Examination.

Regarding COGNISTAT, we found a regional cluster (15, 16) that exhibited a significant positive correlation between GMV and orientation scores in the right IPL. In this ROI, the peak location (MNI coordinate) was *x* = 45, *y* = −48, *z* = 21 (cluster size = 15 voxels; voxel-level FWE corrected, *p* < 0.05, (1-β) = 0.949; Figure 1A). The correlation coefficient (*r*) between GMV in the right IPL and the orientation scores was 0.63 (Figure 1B), which suggested the positive correlation. Although there was no statistically significant difference after adjusting for sex, age, length of education and total MMSE score (FWE corrected, *p* = 0.193, (1-β) = 0.934), there was a statistical significance or a trend toward significance for the adjustment for sex, age, length of education, and total MMSE score, respectively (FWE corrected, *p* < 0.05, (1-β) = 0.947; *p* = 0.054, (1-β) = 0.947; *p* = 0.063, (1-β) = 0.947; *p* < 0.05, (1-β) = 0.947). In contrast, other ROIs (left IPL and bilateral precuneus) did not exhibit a significant positive correlation between GMV and orientation scores. Regarding MMSE, we did not find any significant correlations between GMV and orientation score in any ROI. Finally, we could not find statistically significant differences in any brain regions between converters and non-converters.

## Discussion

MMSE is a frequently used assessment tool for cognitive function (7) that can identify disorientation in early AD and in those at high-risk of cognitive decline (17). COGNISTAT has recently been introduced to evaluate cognitive function because its diagnostic accuracy and clinical utility were better than those for MMSE in a primary care population (18). COGNISTAT includes an orientation subtest for people, besides those for time and space. Thus, we used COGNISTAT as the primarily means to evaluate orientation. To our knowledge, this is the first VBM study to identify core brain regions associated with overall mental disorientation in aMCI.

We found that brain atrophy (lower GMV) was related to greater severity of overall mental disorientation in the right IPL of aMCI patients. This finding partially supports our hypothesis that IPL and/or the precuneus is important for disorientation in aMCI. However, we did not find any significant correlation between disorientation as assessed by MMSE and any of our hypothesized brain regions. These results suggest that COGNISTAT may be superior to MMSE in its ability to identify overall mental disorientation related to brain atrophy.

Only the right IPL showed a significant correlation between GMV and disorientation. Brain regions related to disorientation are mostly localized in the right hemisphere, which partially overlaps with the default mode network (DMN) (19–21). Furthermore, a previous neuropathological study on AD demonstrated that the relationship between disorientation (time and place) and parietal lobe was stronger in the right hemisphere (22). Taken together, the right IPL of aMCI may be more involved in overall mental disorientation than the left IPL.

Recent fMRI study demonstrated that common cortical activity related to orientation in time, space, and to people are precisely localized to the IPL and precuneus (5). This finding partially supports our findings that the IPL plays an important role in disorientation in aMCI. Among aMCI and AD, the distribution of amyloid deposition is remarkably similar to the spatial pattern of the DMN (23). Therefore, the orientation network is probably impaired in aMCI because pathological changes in it were observed in AD (22).

Although a previous SPECT study of AD showed hypoperfusion of the precuneus (24), we could not observe a relationship between GMV in the precuneus and disorientation. Interestingly, a recent diffusion MRI study revealed that the IPL was anatomically connected with the precuneus by short-range bilateral white matter tracts (25). This functional communication between areas is important for processing cognitive functions, including orientation. Accordingly, low GMV in the IPL might induce dysfunction of the precuneus, which results in disorientation in aMCI.

It is interesting to note that glutathione (GSH) serves as a marker of oxidative stress that is an important factor in MCI and AD (26–29). Mandal et al. (27) reported that GSH levels in hippocampus measured by magnetic resonance spectroscopy (MRS) accurately discriminated MCI from healthy individuals and that GSH levels correlated with cognitive function. The parietal cortex has more GSH content than other brain regions in healthy people in MRS (26). Decreased GSH level in the parietal cortex may underlie low orientation scores of aMCI in this study. Thus, the combined use of GSH-MRS and VBM analysis is useful to clarify the neural correlates of disorientation in aMCI.

This study was limited by the relatively small sample size. Particularly, we could not find a positive correlation between the right IPL and orientation scores after adjusting for demographical variables. There was also no significant difference in GMV loss between converters and non-converters. We could not assess the correlation with orientation subtests. Since orientation scores of COGNISTAT does not show normal distribution, it may be smeared by celling effect. Further longitudinal and larger scale VBM studies should be considered to verify the brain regions associated with disorientation in time, space, and to people in aMCI.

In conclusion, the right IPL is responsible for overall mental disorientation in aMCI. Therefore, brain atrophy in the right IPL can be useful for early detection of aMCI.

## Author Contributions

AO, TY, AT, and MM collected data. AO analyzed the data. AO, TY, and ST wrote the manuscript. AO, TY, ST, AT, and MM have read and approved the final version of the manuscript.

### Conflict of Interest Statement

The authors declare that the research was conducted in the absence of any commercial or financial relationships that could be construed as a potential conflict of interest.
